# Dose escalation of accelerated hypofractionated three-dimensional conformal radiotherapy (at 3 Gy/fraction) with concurrent vinorelbine and carboplatin chemotherapy in unresectable stage III non-small-cell lung cancer: a phase I trial

**DOI:** 10.1186/1748-717X-8-201

**Published:** 2013-08-17

**Authors:** Qiang Lin, Yue-E Liu, Xiao-Cang Ren, Na Wang, Xue-Ji Chen, Dong-Ying Wang, Jie Zong, Yu Peng, Zhi-Jun Guo, Jing Hu

**Affiliations:** 1 Department of Oncology, North China Petroleum Bureau General Hospital of Hebei Medical University, 8 Huizhan Avenue, Renqiu, Hebei Province 062552, P.R. China

**Keywords:** Accelerated hypofractionated radiotherapy, Three-dimensional conformal radiotherapy, Non-small-cell lung cancer, Concurrent chemoradiotherapy, Maximum tolerated dose, Vinorelbine, Carboplatin

## Abstract

**Background:**

Accelerated hypofractionated radiotherapy can shorten total treatment time and overcome the accelerated repopulation of tumour cells during radiotherapy. This therapeutic approach has demonstrated good efficacy in the treatment of locally advanced non-small-cell lung cancer (NSCLC). However, the optimal fractionation scheme remains uncertain. The purpose of this phase I trial was to explore the maximum tolerated dose (MTD) of accelerated hypofractionated three-dimensional conformal radiotherapy (3-DCRT) (at 3 Gy/fraction) administered in combination with concurrent vinorelbine (NVB) and carboplatin (CBP) chemotherapy for unresectable stage III NSCLC.

**Methods:**

Previously untreated cases of unresectable stage III NSCLC received accelerated hypofractionated 3-DCRT, delivered at 3 Gy per fraction, once daily, with five fractions per week. The starting dose was 66 Gy and an increment of 3 Gy was utilized. Higher doses continued to be tested in patient groups until the emergence of dose-limiting toxicity (DLT). The MTD was regarded as the dose that was one step below the dose at which DLT occurred. Patients received at least one cycle of a concurrent two-drug chemotherapy regimen of NVB and CBP.

**Results:**

A total of 13 patients were enrolled and progressed through three dose escalation groups: 66 Gy, 69 Gy, and 72 Gy. No treatment-related deaths occurred. The major adverse events included radiation oesophagitis, radiation pneumonitis, and neutropenia. Nausea, fatigue, and anorexia were commonly observed, although the magnitude of these events was typically relatively minor. Among the entire group, four instances of DLT were observed, including two cases of grade 3 radiation oesophagitis, one case of grade 3 radiation pneumonitis, and one case of grade 4 neutropenia. All of these cases of DLT occurred in the 72 Gy group. Therefore, 72 Gy was designated as the DLT dose level, and the lower dose of 69 Gy was regarded as the MTD.

**Conclusions:**

For unresectable stage III NSCLC 69 Gy (at 3 Gy/fraction) was the MTD of accelerated hypofractionated 3-DCRT administered in combination with concurrent NVB and CBP chemotherapy. The toxicity of this chemoradiotherapy regimen could be tolerated. A phase II trial is recommended to further evaluate the efficacy and safety of this regimen.

## Background

Locally advanced non-small cell lung cancer (NSCLC) accounts for approximately 1/3 of NSCLC cases, and challenges remain with respect to the treatment of this form of NSCLC [1]. In the treatment of locally advanced NSCLC, sequential chemoradiotherapy exhibits greater efficacy than radiotherapy alone [2], whereas concurrent chemoradiotherapy exhibits greater efficacy than sequential chemoradiotherapy [3-5]. Therefore, at present, concurrent chemoradiotherapy is the standard treatment for locally advanced NSCLC [1,5].

In particular, compared with sequential chemoradiotherapy, concurrent chemoradiotherapy achieves improved overall survival (OS), producing absolute benefits in 3-year OS and 5-year OS of 5.7% and 4.5%, respectively; this increase in OS may primarily be attributed to improved locoregional control [5]. At present, concurrent chemoradiotherapy for locally advanced NSCLC involves a standard radiation dose of 60 Gy with conventional fractionation. To achieve more effective results, the Radiation Therapy Oncology Group (RTOG) and the North Central Cancer Treatment Group (NCCTG) conducted studies (RTOG 0117 and NCCTG 0028, respectively) to examine dose escalation with conventional fractionation; these phase I/II trials determined that doses of 74 Gy could be tolerated and generated encouraging survival results [6,7]. However, the results of the subsequent phase III randomised controlled study (RTOG 0617) were disappointing and difficult to comprehend. In this trial, less favourable survival outcomes were observed for the 74 Gy high-dose group than for the 60 Gy low-dose group, with one-year OS rates of 70.4% and 81%, respectively; moreover, because preliminary analyses revealed no significant differences in toxicity between the high-dose group and the low-dose group, these results did not reflect excessive treatment toxicity for the high-dose group [8]. NSCLC involves rapidly proliferating cells that exhibit a cell doubling time of only 2.5-3.3 days, and accelerated repopulation occurs during radiotherapy. If the duration of radiotherapy treatments extends for longer than six weeks, each additional day of treatment is associated with a 1.6% decrease in survival [9]. Although the exact reasons why high-dose radiotherapy in the RTOG 0617 study failed to produce survival benefits remain unclear, one potential factor that merits consideration is the long treatment time of 7.4 weeks that is involved in conventional fractionation [1].

Studies have demonstrated that higher biologically effective doses (BEDs) of radiotherapy in cancer treatments will improve local control and survival [10,11]. Because dose escalation with conventional fractionation requires a significant increase in treatment time, two methods to improve BED that maintain or reduce treatment time have been explored: hyperfractionation and hypofractionation. Certain hyperfractionation radiotherapies for NSCLC have achieved good clinical efficacy, including hyperfractionated accelerated radiotherapy (HART) and continuous HART (CHART). However, these therapies not only exhibit acute toxicity but also involve multiple treatments per day that produce inconvenience and more economic costs for patients; thus, it is not convenient for clinical applications of these therapeutic techniques [12,13]. Advances in modern radiotherapy techniques have led to the development of three-dimensional conformal radiotherapy. Compared to prior radiotherapy approaches, three-dimensional conformal radiotherapy can significantly lower the doses of radiation to normal tissues, allowing for the administration of larger doses per fraction on tumors. Accelerated hypofractionated irradiation at 3 Gy per fraction is frequently used for the treatment of locally advanced NSCLC [14-16]; under certain conditions, the dose of radiation used in this approach can be incremented to 75 Gy [16]. To the best of our knowledge, no studies have examined 3 Gy/fraction radiotherapy with concurrent chemotherapy. The purpose of this phase I trial was to determine the maximum tolerated dose (MTD) of accelerated hypofractionated three-dimensional conformal radiotherapy (at 3 Gy/fraction) that is administered in combination with concurrent vinorelbine (NVB) and carboplatin (CBP) chemotherapy for the treatment of unresectable stage III NSCLC.

## Methods

### Eligibility

Patients with previously untreated unresectable stage IIIA or stage IIIB NSCLC (as defined by the 2009 staging standards of the International Union Against Cancer (UICC)) were recruited, who were confirmed pathologically or cytologically. The patients had to be older than 18 years of age and no more than 75 years of age. The Karnofsky performance status (KPS) scores of these patients were at least 70, and the patients were expected to survive for at least 3 months. With respect to laboratory test results, the patients possessed absolute neutrophil counts (ANCs) of at least 2.0×10^9^/L; haemoglobin levels of at least 100 g/L; platelet counts (PLTs) of at least 100×10^9^/L; and serum creatinine, alanine aminotransferase, aspartate aminotransferase, and total bilirubin levels that were below the upper limit of the normal range. No significant electrocardiography (ECG) abnormalities and no requirement hospitalisation for diseases other than NSCLC.

Exclusion criteria: pregnant or breastfeeding; with malignancies other than NSCLC, with the exception of cases of cervical carcinoma in situ or non-malignant melanoma skin cancer that had been clinically cured for at least five years; who could not undergo concurrent chemoradiotherapy for medical reasons; or suffered from either superior vena cava syndrome or severe lung disorders that affected lung function.

This clinical trial was approved by the Ethics Committee of Hebei Medical University. This study was performed in accordance with the standards for human clinical trials and the principles stated in the Declaration of Helsinki (as issued in 1975 and revised in 2000). All patients signed an informed consent form prior to enrolment.

### Patient assessment

All patients underwent medical assessments within 2 weeks prior to the start of treatment that included the acquisition of a complete medical history; a complete physical examination; thoracoabdominal contrast-enhanced computed tomography (CT); CT or magnetic resonance imaging (MRI) of the brain; ECG; and a bronchoscopy. If clinically indicated, a whole-body bone scan was obtained by emission CT (ECT), routine blood testing was performed, and a comprehensive blood biochemical profile was acquired.

Patients underwent a physical examination and routine blood testing each week (with increased examination frequencies as necessary). Full biochemical profiles were obtained and ECG was performed prior to each chemotherapy cycle.

### Study design

This phase I trial was an open-label, non-randomised study of radiation dose escalation. The primary endpoint of the study was the determination of the MTD for accelerated hypofractionated three-dimensional conformal radiotherapy (at 3 Gy/fraction) administered in combination with concurrent NVB and CBP chemotherapy. The secondary endpoints included short-term efficacy and progression-free survival (PFS). Each cohort included at least three cases treated with escalating-dose radiotherapy and fixed-dose chemotherapy; the chemoradiotherapy treatment scheme is depicted in Table 1.

**Table 1:** Concurrent chemoradiotherapy schema

**Concurrent chemoradiotherapy schema**						
**RT regimen: Weeks 1–5: 3 Gy/f, 1 f/d,5 f/w;**					**Dose escalation**	
Week	1	2	3	4	5	6
RT					**Level 1:**	
					**Level 2:**	
					**Level 3:**	
Chemotherapy: NVB (25 mg/m2) d1, d8; CBP,AUC = 5 mg/m1.min on d8, repeated every 28 d						
NVB	◆	◆			◆	◆
**CBP**		●				●

### Radiation therapy

Patients received radiotherapy in the supine position with their hands folded on top of their heads. A vacuum pad was used to immobilise a patient’s body position and appropriately limit respiratory motion. Contrast-enhanced spiral CT (GE Light Speed Plus 4) was performed in the treatment body position. The image data were input into the three-dimensional treatment planning system. The Venus 5014 software package (Shanghai Tuoneng Co., Shanghai, China) was employed to design the radiotherapy plan, using convolution algorithms. The delineation of the target volume was performed in accordance with the consensus guidelines for the delineation of radiotherapy targets in NSCLC [17]; in particular, the target area of the primary lesion was delineated in the routine lung window (1600, -600 Hounsfield units (HU)), and the mediastinal target area was delineated in the mediastinal window (400, 20 HU). The treatment regimen utilised involved-field radiation without elective nodal irradiation. Target volumes were defined as follows: the gross tumour volume (GTV) was defined as the primary lesion and lymph nodes with short diameters of greater than 1 cm in CT imaging; the clinical target volume (CTV) was defined as the GTV enlarged by margins of 6 mm (in cases of squamous cell carcinoma and metastatic lymph nodes) or 8 mm (in cases of adenocarcinoma); the planning target volume (PTV) was defined as the CTV enlarged by a margin of 10 to 15 mm based on the respiratory movement observed in the simulator. The GTV was confirmed by two radiation oncologists and one diagnostic imaging specialist. The radiation oncologists outlined vital organs and body surface contours. Three to six coplanar or non-coplanar fields were utilised for conformal radiation. Dose-volume histograms (DVHs) were used to optimise the therapeutic plan. The following limiting conditions for the radiation received by vital organs were employed: the maximum V20 (the percentage of healthy organ volume that receives 20 Gy radiation) for both lungs was 30%; 0% of the oesophagus was permitted to receive more than 70 Gy of radiation; a maximum of 10 cm of the oesophagus was permitted to receive 60 Gy or more of radiation; 0% of the spinal cord should receive more than 40 Gy of radiation; and the maximum V40 for the heart was 40% [18]. The treatment utilised 6 MV X-rays from a Siemens Primus Plus linear accelerator that was equipped with a 27-pair multi-leaf collimator (Topslane@_M, Shanghai Tuoneng Co., Shanghai, China). All lesions were located in a single target area if possible, and a second target area was established to address distant lesions.

The full course of accelerated hypofractionated radiotherapy was conducted at 3 Gy/fraction, once daily, with five fractions per week; this radiotherapy regimen was completed in 4.4-5 weeks.

### Chemotherapy

Chemotherapy began on the first day of radiotherapy. NVB was administered by intravenous infusion at a dose of 25 mg/m^2^ on day 1 (d1) and day 8 (d8). CBP was administered at an area under the concentration-time curve (AUC) of 5 mg/ml on d8. This treatment was repeated every 28 days. At least one cycle of chemotherapy was performed concurrently with radiotherapy. After radiotherapy had concluded, patients received a maximum of four cycles of consolidative chemotherapy, utilising the same chemotherapy regimen that was employed during the concurrent chemoradiotherapy [18]. Anti-emetics, hepatoprotective drugs, and other treatments were also administered.

### Supportive care

To ensure the implementation of the chemoradiotherapy regimen, patients whose ANCs decreased to less than 2.0 × 10^9^/L were treated with granulocyte colony-stimulating factor (GSF) until their ANCs had increased to at least normal levels. Patients with PLTs of less than 75 × 10^9^/L were treated with interleukin-11 until PLTs had increased to at least normal levels. Nutritional support via intravenous rehydration was also provided to patients as necessary.

### Dose escalation and determinations of dose-limiting toxicity (DLT)

A limited number of reports address hypofractionated radiotherapy with concurrent chemotherapy, and only the European Organisation for Research and Treatment of Cancer (EORTC) has performed a phase I study of this therapeutic approach. This EORTC study determined that this type of chemoradiotherapy approach should be safe at a radiation dose of 66 Gy with fractions of 2.75 Gy; however, this investigation did not report an MTD and used low doses of cisplatin alone as a chemotherapy regimen [19]. In our previous studies of concurrent chemoradiotherapy for lung cancer and oesophageal cancer, we demonstrated that Eastern and Western patient populations exhibit different tolerances to chemoradiotherapy [18,20,21]. In examinations of three-dimensional conformal radiotherapy with conventional fractions of 2 Gy/f in combination with concurrent NVB and CBP chemotherapy, we found that radiation doses of 70 Gy could be tolerated [18,21]. For this study, we therefore arbitrarily chose 66 Gy as a starting radiotherapy dose with a 3 Gy increment. A modified Fibonacci scheme was utilised for dose escalation [18,22], and every cohort contained at least 3 patients. If no dose-limiting toxicity (DLT) was observed after the completion of full-dose radiotherapy and at least concurrent one cycle chemotherapy, the next dose level was applied. However, repeated administration to the same patient was not allowed. If 1 of 3 patients treated within a dose level experienced a DLT, 3 additional patients were treated at the same level. If a second case of DLT was observed, the dose escalation was stopped, and the tested dose was defined as the level of DLT. The MTD was defined as the dose level below the dose that produced the DLT. Acute toxicity was monitored for all three cases in each dose group for the first 90 days following the start of radiotherapy.

Treatment toxicity was evaluated in accordance with the Common Terminology Criteria for Adverse Events (CTCAE), version 3.0, which have been issued by the National Cancer Institute/National Institutes of Health (NCI/NIH). Weekly assessments of toxicity occurred during the concurrent chemoradiotherapy treatment. DLT was defined as a severe and/or life-threatening adverse event that influenced the implementation of concurrent chemoradiotherapy. These adverse events included radiation pneumonitis of grade 3 or higher, oesophagitis of grade 3 or higher, and other non-haematologic toxicities of grade 3 or higher (with the exception of grade 3 nausea, vomiting, fatigue, and/or weight loss); grade 3 febrile neutropenia; grade 4 neutropenia; grade 3 or grade 4 thrombocyotpenia; grade 3 or grade 4 anaemia; and any grade 5 adverse effects [18].

### Evaluations of short-term tumour treatment efficacy

Evaluations of short-term treatment efficacy were performed by examining thoracoabdominal spiral CT results at four weeks after the completion of radiotherapy; the Response Evaluation Criteria in Solid Tumours, version 1.1 (RECIST 1.1), standard was utilised for these assessments [23].

### Dose attenuation

Dose attenuations were implemented based on the most serious adverse events that occurred at any point after the start of treatment.

Because we were conducting a radiotherapy dose escalation study, no reductions in radiation dose were permitted. However, if toxicity of grade 3 or higher occurred (with the exception of grade 3 nausea, vomiting, or weight loss), radiotherapy was postponed until the toxicity had been resolved. In contrast, if adverse events occurred that were unrelated to radiotherapy, such as peripheral neuropathy, the radiotherapy was continued but chemotherapy was suspended. The chemotherapy was resumed after these adverse events had dissipated.

The following chemotherapy dose attenuation procedures were employed. In the event of grade 3 or grade 4 thrombocytopenia, grade 3 or grade 4 anaemia, grade 4 neutropenia, or grade 3 or grade 4 non-haematologic toxicities (except for grade 3 nausea, vomiting or weight loss), both radiotherapy and chemotherapy were suspended until the toxicity had been resolved. If the toxicity had not been resolved within two weeks, the patient was withdrawn from the study. The NVB and CBP doses of this patient’s subsequent chemotherapy cycle were reduced by 25%, and the patient received prophylactic GSF treatment. If a patient exhibited grade 3 neutropenia or grade 2 thrombocytopenia, chemotherapy was stopped but radiotherapy was continued. The NVB and CBP doses of this patient’s subsequent chemotherapy cycle remained unchanged, and the patient received prophylactic GSF treatment [18].

### Follow-up and statistics

Follow-up was conducted every three months for the first two years after the completion of radiotherapy and every six months thereafter. All statistical analyses were performed using the SPSS 19.0 biostatistical software package. The survival data were evaluated using the Kaplan-Meier method. The survival time was measured from the initiation of the concurrent chemoradiotherapy until death due to any cause or the last follow-up. Only the initial instance of treatment failure was considered with respect to assessing causes of treatment failure. PFS was defined as survival without local recurrence or distant metastases.

## Results

### Patients characteristics

From August 2010 to October 2012, 13 patients with pathologically or cytologically confirmed cases of previously untreated NSCLC were enrolled into this study. All 13 patients underwent toxicity and efficacy evaluations. The patient characteristics are provided in Table 2. The median age of was 65 years, and their median KPS score was 80. The 13 cases included seven cases of squamous cell carcinoma and 6 cases of adenocarcinoma. There were six cases of stage IIIA cancer and seven cases of stage IIIB cancer. The median GTV was 84.6 cm^3^ (with a mean of 97.2 cm^3^ and a range of 22.3-222.5 cm^3^), and the median PTV was 234.9 cm^3^ (with a mean of 242.7 cm^3^ and a range of 111.6-369.7 cm^3^).

**Table 2:** Patient characteristics

**Characteristic**		**No. of patients**		**Percentage of Patients (%)**
		**N = 13**		
Gender				
	Male	9		69.2
	Female	4		30.8
Age				
	Median		65	
	Range		57-75	
Karnofsky performance status				
	Median		80	
	Range		70-90	
Histology				
	Squamous cell carcinoma	7		53.8
	Adenocarcinoma	6		46.2
Stage				
	IIIA	6		46.2
	IIIB	7		53.8
GTV				
	Median		84.6 cm^3^	
	Range		22.3-222.5 cm^3^	
PTV				
	Median		234.9 cm^3^	
	Range		111.6-369.7 cm^3^	

### Compliance

All patients received escalating-dose radiotherapy at one of the three dose levels of 66 Gy, 69 Gy, and 72 Gy; in particular, three, six, and four patients, respectively, were enrolled at these dose levels, as presented in Table 3. All of the patients completed the radiotherapy dose and received at least one cycle of concurrent chemotherapy. All 13 patients received consolidative chemotherapy with a median of four chemotherapy cycles (with a range of one to four cycles).

**Table 3:** Radiation dose escalation schema

**Dose level**	**3D**-**CRT hypo (Gy)**	**Cases**
1	66	3
2	69	6
3	72	4

### Non-haematologic toxicity

Radiation pneumonia and radiation oesophagitis were commonly observed, although the majority of cases of these toxicities were mild. Among the whole group, there was only one case of grade 3 radiation pneumonitis and two cases of grade 3 radiation oesophagitis. Nausea, vomiting, fatigue, and anorexia were observed in most of cases; however, these adverse effects were mild and were successfully alleviated without affecting the implementation of chemoradiotherapy through the administration of appropriate antiemetics and intravenous rehydration. Liver and kidney toxicity was rare. Detailed information regarding the non-haematologic toxicities is provided in Table 4.

**Table 4:** Nonhematologic toxicity

**Toxicity**	**66 Gy**	**69 Gy**	**72 Gy**	**Total**
	**Case**	**Case**	**Case**	**Case (%)**
Radiation pneumonia				10(76.9)
I	1	2	1	
II	1	2	2	
III	0	0	1	
Radiation oesophagitis				11(84.6)
I	1	2	1	
II	1	3	1	
III	0	0	2	
Radiation dermatitis				9(69.2)
I	1	3	3	
II	0	1	1	
Nausea				12(92.3)
I	1	2	1	
II	1	2	1	
III	1	1	2	
Vomiting				8(61.5)
I	1	1	2	
II	1	1	1	
III	0	1	0	
Anorexia				12(92.3)
I	1	1	1	
II	2	2	1	
III	0	2	2	
Fatigue				11(84.6)
I	0	3	1	
II	1	2	2	
III	1	0	1	
ALTI	0	1	2	3(23.1)
ASTI	1	0	2	3(23.1)
Cr I	1	0	1	2(15.4)
BIL I	0	1	0	1(7.7)

ALT: alanine aminotransferase; AST: aspartate aminotransferase; Cr: serum creatinine; BIL: bilirubin.

### Haematologic toxicity

All patients exhibited neutropenia; in particular, 76.9% of cases exhibited grade 1 or grade 2 neutropenia, only 15.3% of the patients exhibited grade 3 neutropenia, and only one patient exhibited grade 4 neutropenia. Thrombocytopenia and anaemia were primarily observed at the grade 1 or grade 2. The haematologic toxicities observed in this study are detailed in Table 5.

**Table 5:** Hematologic toxicity

**Toxicity**	**66 Gy**	**69 Gy**	**72 Gy**	**Total**
	**Case**	**Case**	**Case**	**Case (%)**
Neutropenia				13(100)
I	1	2	1	
II	2	2	2	
III	0	2	0	
IV	0	0	1	
Thrombocytopenia				8(61.5)
I	1	2	1	
II	1	1	2	
Anemia				8(61.5)
I	1	3	2	
II	1	0	1	

### The determination of the MTD

Three patients were initially enrolled into the 66 Gy dose group, and none of these patients experienced DLT. Similarly, three patients were initially enrolled into the 69 Gy dose group, and none of these patients experienced DLT. In contrast, three patients were initially enrolled into the 72 Gy dose group, and all of these patients experienced DLT. In particular, one patient exhibited grade 3 radiation pneumonitis, one patient exhibited grade 3 radiation oesophagitis, and one patient exhibited grade 4 neutropenia. To confirm these findings, the enrolment of three additional patients at the 72 Gy dose level was planned; however, the single patient who was first enrolled to achieve this purpose experienced a DLT involving grade 3 radiation oesophagitis. Moreover, adverse reactions occurred more frequently in the 72 Gy group than in the remaining dose groups; this group accounted for 52.9% (9/17) of the grade 3 or grade 4 adverse reactions of the entire study but only 30.8% (4/13) of the patients of the study. However the occurrence of the grade 3 or grade 4 adverse effects in the 66 Gy and 69 Gy dose level was 47.1% (6/17) of the entire study but 69.2% (9/13) of the patients of the study. These findings suggested that the 72 Gy dose level could not be tolerated; as a result, no additional patients were enrolled at the 72 Gy dose level. To confirm that the 69 Gy dose level was safe, an additional three patients were enrolled at the 69 Gy dose level, and none of these patients experienced DLT.

In accordance with the study design, because the 72 Gy dose level produced severe toxicities, including all four patients at this dose level experiencing DLT, 72 Gy was designated as the dose level at which DLT occurred; thus, 69 Gy, as the highest tested dose that was less than 72 Gy, was regarded as the MTD.

### Short-term treatment efficacy

Evaluations of the short-term treatment efficacy among the 13 study participants revealed that 23.1% (3/13) of participants exhibited a complete response (CR), 61.5% (8/13) of participants exhibited a partial response (PR), 15.4% (2/13) of participants exhibited a stable disease (SD) state, and no cases exhibited a progressive disease (PD) state. The total short-term response rate (RR) was therefore 84.6% (11/13).

### Survival

**Figure 1:**
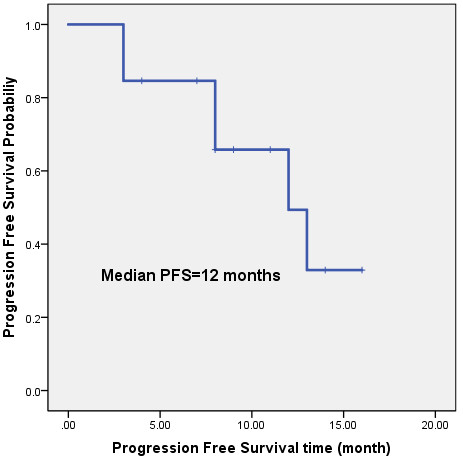
**The median PFS time was 12 months, and 1- year PFS rate 49.4%.**

Although this study is merely a phase I trial, we nonetheless determined preliminary survival results. Because of the short follow-up period (from four to 20 months), which included a median follow-up period of 10 months, only three deaths occurred; however, mature OS data have not yet become available. The median PFS was 12 months, and 49.4% of 1-year PFS (Figure 1). Recurrence or progression occurred in six of the examined cases, including one case of recurrence within the radiation field, one case involving recurrence within the radiation field and distant metastasis (liver metastasis), and four cases of distant metastases (two cases of brain metastasis, one case of liver metastasis and bone metastasis, and one case of adrenal metastasis).

## Discussion

Radiotherapy is one of the most important treatments for NSCLC, and radiation dose levels significantly affect treatment outcomes. Fletcher [24] believed that a radiotherapy dose of 80–100 Gy was required to cure lung cancer. This assume has been confirmed through a clinical trial of stereotactic body radiotherapy, which indicated that significantly higher survival rates were observed among patients who were treated with a BED of at least 100 Gy than among patients who were treated with a BED of less than 100 Gy [25]. Relative to traditional radiotherapy approaches, the techniques of three-dimensional conformal radiotherapy and intensity-modulated radiotherapy can significantly reduce the radiation doses that are received by normal tissues and organs; in the context of radiotherapy alone (without accompanying chemotherapy), these techniques allow tolerable toxicity to be achieved even at radiation doses as high as 83.3 Gy or 103 Gy [26,27]. NSCLC cells demonstrate accelerated repopulation during radiotherapy, and increases in treatment duration can therefore reduce local control, negatively impacting survival. Thus, to improve local control it is critical to achieve higher BEDs at constant or reduced overall treatment durations [9,28]. However, hyperfractionated radiotherapy not only produces more severe acute reactions than conventional radiotherapy but also requires three treatments per day, producing inconvenience and much more treatment costs for patients; thus, it is not easy to clinically utilise hyperfractionated radiotherapies [12,13].

Mehta et al. [9] suggested that for cancers with very short cell doubling times, such as NSCLC, increasing the dose per fraction constitutes a promising method of achieving dose escalation. In cases of stage I/II NSCLC, hypofractionated stereotactic radiotherapy has achieved effects comparable to the results of surgery; in these cases, the radiotherapy regimen includes extremely high doses of up to 18–22 Gy per fraction [29,30]. However, in cases of locally advanced NSCLC, because of concerns regarding toxicities (particularly late toxicities) to the oesophagus, trachea, ribs, nerves, and other vital organs, it has not been possible to achieve these high doses per fraction [30]. In fact, the appropriate dose for hypofractionated radiotherapy for locally advanced NSCLC remains unclear [1].

Thirion et al. [31] conducted a study of accelerated hypofractionation using 3 Gy per fraction in which a 72 Gy radiation dose was delivered in 24 fractions over five weeks. The 25 NSCLC cases that Thirion et al. examined included 16 cases of stage III NSCLC. In the investigation conducted by Thirion et al., one case experienced grade 3 radiation pneumonitis, two experienced grade 3 radiation oesophagitis, and no grade 4 or higher adverse events occurred among the entire group. Xie et al. [16] performed a dose escalation trial of accelerated hypofractionated radiotherapy with 3 Gy increments using cases from the Chinese population and found that patients could tolerate the radiotherapy treatment at doses of up to 75 Gy at V20 levels of no greater than 20% or at doses of up to 69 Gy at V20 levels of between 20% and 30%. The Fudan University Cancer Centre reported the results of a Phase II trial of accelerated hypofractionated radiotherapy with sequential chemotherapy for NSCLC [32]. In this trial, the initial radiation dose was 50 Gy with 2.5 Gy fractions; the dose was subsequently increased at 3 Gy/fraction to a total radiation dose of 65 to 68 Gy, and elective nodal irradiation was not performed. All patients received 2 cycles of induction chemotherapy (with full-dose NVB and cisplatin), and good treatment efficacy was observed. The median PFS, median OS, and three-year OS were 10 months, 19.0 months, and 32.1%, respectively, with acceptable treatment toxicities.

Conventional fractionated radiotherapy with concurrent chemotherapy achieves superior efficacy to sequential chemoradiotherapy; moreover, experimental studies have demonstrated that hypofractionated radiotherapy combined with chemotherapy can achieve significantly increased biological effects [33]. Therefore, compared with these treatments, accelerated hypofractionated radiotherapy combined with concurrent chemotherapy should theoretically be capable of producing additional increases in therapeutic efficacy. However, due to concerns that hypofractionated radiotherapy with concurrent chemotherapy could have aggressive toxicity, little research has been conducted in this area. In addition, investigations of this topic have utilised very different fractionation regimens and concurrent chemotherapy drugs. In particular, the studied doses per fraction have ranged from 2.4 Gy to 2.75 Gy, and the concurrent chemotherapy drugs have included low daily doses of cisplatin, weekly paclitaxel/CBP, and full-dose NVB/cisplatin [19,34-36].

An EORTC study reported dose escalation results from hypofractionation radiotherapy with 2.75 Gy fractions for NSCLC and suggested that radiotherapy with a total dose of 66 Gy in 24 fractions in combination with concurrent low-dose cisplatin could be well tolerated [19]. Based on these results, a phase III randomised study was conducted to compare concurrent chemoradiotherapy with sequential chemoradiotherapy [34]. Because this study administered elective nodal irradiation, high rates of severe acute oesophagitis were observed among the concurrent group; in particular, grade 3 and grade 4 acute oesophagitis was observed in 14% and 3%, respectively, of the patients in concurrent group, whereas significantly lower rates of acute oesophagitis were observed in the sequential group. Interestingly, rates of late oesophageal injury were not significantly higher in the concurrent group (5%) than in the sequential group (4%). The OS did not differ between the two groups; in particular, the concurrent group and the sequential group exhibited 2-year OS of 39% and 34%, respectively, and three-year OS of 34% and 22%, respectively. This lack of improvement in OS with the concurrent therapy compared to the sequential therapy may relate to the low doses of chemotherapy that were administered in this study. A 2011 report of the American Society of Clinical Oncology (ASCO) discussed the preliminary results of a British study of radiotherapy with concurrent two-drug chemotherapy for NSCLC, using a total radiation dose of 55 Gy in 20 fractions with 2.75 Gy/fraction; in particular, this treatment produced a median survival time (MST) of 27.4 months, a 2-year OS of 54%, and a 3-year OS of 38% [35]. In this study, full-dose of chemotherapy drugs were administered in a regimen that included 15 mg/m^2^ NVB on d1, d6, d15, and d20 as well as 20 mg/m^2^ cisplatin on d1-4 and d16-19 [36]. A phase II trial by the Korean Radiation Oncology Group also achieved good survival results, including an MST of 28.1 months and 2- and 3-year OS of 56.4% and 43.8%, respectively [37]. This study used a radiotherapy regimen consisting of a total radiation dose of 60 Gy in 25 fractions of 2.4 Gy/fraction, which was combined with concurrent paclitaxel and CBP chemotherapy. Elective nodal irradiation was not performed, and tolerable toxicity was observed.

The aforementioned three regimens of hypofractionated radiotherapy with concurrent chemotherapy involved significant differences in radiotherapy fractionation, total radiation dose, and concurrent chemotherapy protocols. In addition, distinct inclusion criteria were used in these studies, and different restrictions on the radiation dose received by normal tissues and organs were employed. Thus, it is difficult to horizontally compare the advantages and disadvantages of these prior investigations to identify a regimen that could receive widespread acceptance. However, it is evident that the efficacy of hypofractionated radiotherapy with concurrent chemotherapy exhibits good comparability with the efficacy of conventional fractionation with concurrent chemotherapy [3-5].

In our trial, we chose 3 Gy as the dose per fraction because the use of hypofractionated radiotherapy at 3 Gy per fraction was a relatively mature approach for radiation treatment alone; in addition, the chosen radiotherapy regimen was efficient and was expected to be completed in a total treatment time of five weeks. Thus, this regimen could achieve a high rate of tumour control [9]. This phase I trial revealed that hypofractionated radiotherapy at 3 Gy/fraction with concurrent full-dose chemotherapy exhibited high levels of safety. No treatment-related deaths occurred during the entire study. The main toxicities that were observed included radiation oesophagitis and radiation pneumonitis. The rate of radiation oesophagitis reached 84.6% (11/13) in whole group. Because we utilised the involved-field irradiation technique and refrained from administering elective nodal irradiation, the observed cases of radiation oesophagitis were mainly of grade 1 or grade 2 (69.2%, 9/13). The adverse events to the radiotherapy were tolerable after general symptomatic treatment and did not affect the implementation of the radiotherapy regimen. The 2 observed cases of grade 3 radiation oesophagitis occurred at the 72 Gy dose level. One case of grade 3 radiation pneumonitis occurred in the 72 Gy group, whereas the other observed cases of radiation pneumonitis in this study were all grade 1 or grade 2. Other non-haematologic toxicities were easily managed through clinical treatment and did not affect the implementation of concurrent chemoradiotherapy. Although all patients suffered from neutropenia, most of the cases of these adverse events were mild and did not affect the implementation of concurrent chemoradiotherapy. Only one case of grade 4 neutropenia occurred in this study, and this case arose in the 72 Gy group. In this case, after the administration of treatments to prevent infection and GSF to enhance white blood cell counts, agranulocytosis was resolved within one week, and no serious consequences were observed. Mild degrees of anaemia and thrombocytopenia were observed in the whole group. The short-term tumour control rate of 84.6% indicated that our regimen of hypofractionated radiotherapy with concurrent chemotherapy was able to achieve good local tumour control. Because of the short follow-up time and the small number of examined cases, mature OS data for this study are not yet available; however, the 12-month PFS of this investigation is highly comparable to the 12-month PFS results from other studies [32].

## Conclusions

Accelerated hypofractionated three-dimensional conformal radiotherapy (at 3 Gy/fraction) combined with concurrent NVB and CBP chemotherapy is feasible and safe for the treatment of unresectable stage III NSCLC. The MTD of the radiotherapy was 69 Gy in 23 fractions; at this dose, adverse events could be tolerated. Based on this phase I trial, a phase II trial of a hypofractionated radiotherapy approach involving a total radiation dose of 69 Gy in 23 fractions with 3 Gy/fraction that is administered in combination with concurrent chemotherapy is ongoing to further evaluate the efficacy and safety of this treatment regimen.

## Abbreviations

NSCLC: Non-small cell lung cancer; hypo: Hypofractionated radiation therapy; 3D-CRT: Three-dimensional conformal radiation therapy; DLT: Dose-limiting tolerance; MTD: Maximum-tolerated dose; NVB: Vinorelbine; CBP: Carboplatin; KPS: Karnofsky performance status; CR: Complete response; PR: Partial response; SD: Stable disease; PD: Progressive disease.

## Competing interests

The authors declare that they have no competing interests.

## Authors’ contributions

QL was the PI of this clinical trial, who designed the subject and drafted the manuscript. YL participated in the subject of design and the manuscript writing. XR participated in the design of the subject and carried out the clinical implementation of the study. NW carried out the clinical implementation of the study. XC carried out the clinical implementation of the study. DW guided the design of the subject and helped to draft the manuscript. JZ carried out the clinical implementation of the study. YP carried out the clinical implementation of the study. JG carried out the clinical implementation of the study. JH carried out the clinical implementation of the study. All authors read and approved the final manuscript.

## Authors' information

QL, the corresponding author, is the Associate Professor of Department of Oncology, North China Petroleum Bureau General Hospital of Hebei Medical University, 8 Huizhan Avenue, Renqiu City, Hebei Province, and P.R.China. He is focusing on the chemoradiotherapy on the thoracic neoplasm. He has found difference tolerance between Asian patients and Western patients when they received concurrent chemoradiotherapy in lung cancer and esophageal carcinoma.
